# The role and implication of autophagy in cholangiocarcinoma

**DOI:** 10.1038/s41420-023-01631-7

**Published:** 2023-09-04

**Authors:** Hayat Khizar, Yufei Hu, Yanhua Wu, Jianfeng Yang

**Affiliations:** 1 Department of Gastroenterology, Affiliated Hangzhou First People’s Hospital, Zhejiang University School of medicine, 310006 Hangzhou, Zhejiang China; 2 Department of Oncology, The Fourth Affiliated Hospital, International Institute of Medicine, Zhejiang University School of Medicine, Hangzhou, China; 3 Department of Gastroenterology, The Fourth School of Clinical medicine, Zhejiang Chinese Medical University, Hangzhou, Zhejiang China; 4 Key Laboratory of Clinical Cancer Pharmacology and Toxicology Research of Zhejiang Province, 310006 Hangzhou, Zhejiang China; 5 Key Laboratory of Integrated Traditional Chinese and Western Medicine for Biliary and Pancreatic Diseases of Zhejiang Province, 310006 Hangzhou, Zhejiang China; 6 Hangzhou Institute of Digestive Diseases, 310006 Hangzhou, Zhejiang China

**Keywords:** Targeted therapies, Bile duct cancer

## Abstract

Cholangiocarcinoma (CCA) is a malignant tumor that originates from the biliary epithelial cells. It is characterized by a difficult diagnosis and limited treatment options. Autophagy is a cellular survival mechanism that maintains nutrient and energy homeostasis and eliminates intracellular pathogens. It is involved in various physiological and pathological processes, including the development of cancer. However, the role, mechanism, and potential therapeutic targets of autophagy in CCA have not been thoroughly studied. In this review, we introduce the classification, characteristics, process, and related regulatory genes of autophagy. We summarize the regulation of autophagy on the progression of CCA and collect the latest research progress on some autophagy modulators with clinical potential in CCA. In conclusion, combining autophagy modulators with immunotherapy, chemotherapy, and targeted therapy has great potential in the treatment of CCA. This combination may be a potential therapeutic target for CCA in the future.

## Facts

- Role of autophagy in the progression of CCA.
- Autophagy modulators with clinical potential in CCA.
- Combining autophagy modulators with immunotherapy, chemotherapy, and targeted therapy in the treatment of CCA.

## Open questions

- What’s future use of autophagy in the treatment of CCA?
- How will autophagy work for CCA?
- What will be the impact of combined therapy.

## Introduction

Cholangiocarcinoma(CCA), arising from the biliary epithelium, is the most common bile duct malignancy and the second most common liver cancer after hepatocellular carcinoma (HCC) accounting for 10–20% of all primary liver cancers [1–3]. Fluke infection, inflammatory bowel disease, intrahepatic bile duct stones, choledochal cysts, and primary sclerosing cholangitis are well-known risk factors for CCA. Radical surgical resection is the only effective treatment for early-stage CCA [4, 5]. With the progression of the disease, chemotherapy is one of the effective treatments for advanced CCA. However, chemotherapy has poor inhibition effect on advanced malignant bile duct cells, and the 5-year survival rate is even less than 5% [6, 7].

Autophagy was first proposed by Ashford and Porter in 1962. It refers to the formation of autophagosome by the double-layer membrane covering part of the cytoplasm and the organelles, proteins and other components that are decomposed inside the cell by falling off from the free area of the rough endoplasmic reticulum and fusion with lysosome to form autophagolysosome to degrade the contents of the cell for its own metabolic needs and the renewal of specific organelles. It is usually a survival mechanism that maintains nutrient and energy homeostasis and eliminates intracellular pathogens. However, since the process has multi-steps conditions and control points, once several sites are out of control, it can lead to various human diseases, including cancer [8]. Recent studies have shown that autophagy plays a variety of pathophysiological roles in diseases such as cancer, autoimmune diseases and infection-related diseases. For example, it can help cells clear damaged proteins and pathogens. As a double-edged sword, autophagy can be used as both tumor promoter and tumor suppressor in the occurrence and development of cancer. BNIP3, an autophagy signaling protein (a pro-apoptotic member of Bcl-2), is expressed at high levels in colorectal and gastric epithelial cancers, suggesting that increased expression of BNIP3 may be necessary for the development of these cancers [9]. Beclin1 allele deletion has been found in human breast cancer [10] and prostate cancer [11]. In mouse models, lack of Beclin1 gene is more likely to develop lymphoma, liver cancer and other tumors [12]. In summary, autophagy regulation has emerged as a promising strategy for cancer treatment [13–17].

## Autophagy

### Classification of autophagy

Autophagy can be divided into three types: micro-autophagy (MI), chaperone-mediated autophagy (CMA), and macro-autophagy(M). MI refers to the process in which lysosomes or vacuole intima directly invaginate and wrap intracellular substances, and degrade in the lumen of organelles after membrane rupture [18]. CMA is highly selective and often degrades target proteins with uniquely recognized pentapeptide motifs (KFERQ) by means of cytoplasmic chaperone Hsc70. The receptor LAMP2A on the lysosome membrane recognizes the KFERQ group exposed to the binding protein and transfers the selected protein to the lysosome for degradation [19]. By contrast, MA is an evolutionarily conserved metabolic process. Proteins and other cytoplasmic components are sequestered in an isolation membrane, which expands and closes to form a double-layer vesicle structure, namely autophagosome. Then the autophagosome and lysosome fuse and eventually degrade. The typical process of MA is triggered by the inactivation of the mechanistic target of the mammalian target protein of the rapamycin complex 1 (mTORC1) protein complex. Under physiological conditions, MA can serve as a protective mechanism to maintain the stability of the genome under stress conditions. Then once malignant changes occur, MA will in turn protect tumor cells, maintain their proliferation, metastasis and drug resistance [13, 20–22].

### Process of autophagy

**Fig. 1:**
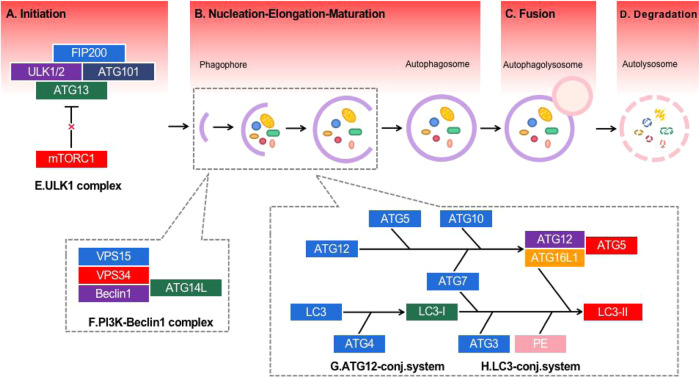
Schematic overview of autophagy. **A** Initiation: ULK1 complex regulates autophagy initiation. **B** Nucleation: The activated ULK1 complex was transferred to phagocytes and activated PI3K-Beclin1 complex to induce vesicle nucleation; Elongation: The cytoplasm and organelles are enveloped and engulfed during elongation; Maturation: Completion and transport of autophagosomes. **C** Fusion: Autophagosomes fuse with lysosomes to form autophagolysosomes. **D** Degradation: Cytoplasmic components are degraded by lysosomal hydrolases. **E** ULK1 complex including ULK1, ULK2, ATG13, FIP200, and ATG101. **F** PI3K-Beclin1 complex including Beclin1, VPS34, VPS15, and ATG14L. **G** The ATG12-conjugation including ATG12, ATG7, ATG10, ATG5, and ATG16L. **H** The LC3-conjugation including LC3, ATG4, LC3-I, ATG7, ATG3, PE, and LC3-II.

The whole process is highly regulated by a limited number of autophagy-related genes (ATGs) [23–25], which can be activated under a variety of stress conditions, such as starvation [26, 27], hypoxia [28, 29], infection, oxidative stress [30, 31], etc. Initiation, nucleation, fusion with lysosomes, and cargo degradation are four stages of process of autophagy [32]. In mammalian cells, the initiation of autophagy is regulated by the Unc-51-like kinase 1 (ULK1) complex, a complex of ULK1, ULK2, ATG13, FIP200 and ATG101. MTOR kinase is a major regulator of this process. Under nutrient-rich conditions, mTOR can phosphorylate ULK1/2 and block the activity of ULK1 complex. When nutrition, energy deficiency or other stress conditions, mTOR activity decreases after inhibition, and ULK1 complex is dephosphorylated and activated. In the autophagy nucleation step, after the activated complex is transferred to phagocytes, the PI3K-Beclin1 complex is activated and vesicle nucleation is induced [33]. During this period, two independent ubiquitin-like binding systems play a key role in the elongation and maturation of autophagy membranes. One is the ATG12-conjugation system. ATG12 is first activated by E1-like enzyme ATG7 [34], which is coupled with ATG5 [35] by E2-like enzyme ATG10 [36] to generate ATG5-ATG12 complex. The complex then further interacts with ATG16L to form ATG16L complex. The other is the LC3-conjugation system. ATG4 cleaves LC3 to produce LC3-I (soluble form). It then binds to phosphatidylethanolamine (PE) via ATG3 and ATG7 [35] to form LC3-II (present on the autophagosome membrane). LC3-II is crucial to the expansion and completion of the autophagy membrane. It is often used as a marker of autophagy progress in research. Then, mature autophagosomes fuse with lysosomes to form autophagic lysosomes. At last, the sequenced cytoplasmic components are degraded by lysosomal hydrolases. The degradation products are recycled to the cytoplasm for cell reuse. The adaptor protein sequestosome 1 (p62) can target specific products of autophagosomes and degrade with other cargo proteins, which is commonly used to measure autophagy flow [35] (Fig. 1).

## The role of autophagy in CCA

CCA is a highly heterogeneous malignant tumor. Many risk factors can lead to chronic inflammation and/or cholestasis, which can alter the heredity and epigenetic inheritance of bile duct cells and ultimately lead to malignant transformation of bile duct cells [37]. Further research on CCA heredity and epigenetic inheritance, CCA occurrence, drug resistance, metastasis and recurrence mechanism will provide more specific therapies for CCA in the future.

Based on anatomic location, CCA are classified as intrahepatic (iCCA), perihilar (pCCA), or distal (dCCA) [38]. Various high-throughput spatial sequencing techniques showed that CCA at different anatomical sites had different molecular profiles and demonstrated heterogeneity of different subtypes. Studies have divided the genome of iCCA into two categories: one is inflammation and the other is proliferation. The former is mainly the activation of inflammatory signals, while the latter is the activation of oncogenic signaling pathways and oncogenes [39]. Most cancers have at least one driver gene mutation, and KRAS is the most common mutation, especially in dCCA [40].

Studies have found that chronic inflammation, epithelial mesenchymal transformation and epigenetic abnormalities are the driving factors of bile duct cell deterioration and play an important role in the progression of CCA. Autophagy is particularly important among these drivers.CCA, with its highly fibrous stroma, is the archetype of inflammatory cancer. Malignant transformation of bile duct cells is associated with chronic cholangitis [41]. Recent studies have found that pro-inflammatory cytokines such as IL-6, endotoxin and TNF are overexpressed in chronic bile duct inflammation. Inflammatory bile duct cells and overexpression of IL-6 contribute to malignant transformation of bile duct cells. During cancer formation, chronic inflammation is involved in a number of signaling pathways that influence the process of autophagy. Qi et al. reported that exposure of human lung epithelial cells to arsenic resulted in cell malignancy, resulting in persistent overexpression of IL6 and inhibition of autophagy [42]. IL6 affects the STAT3 signaling pathway by inhibiting the Beclin1-Bcl-2 complex, leading to carcinogenesis. On the contrary, overexpression of Beclin-1 can enhance autophagy and prevent IL-6-mediated cell malignancy. This correlation between IL6-mediated cellular malignancy and autophagy activity during tumorigenesis may provide a novel therapeutic approach for inflammatory iCCA.

Epithelial-to-mesenchymal transition (EMT) has been suggested as a driver of epithelial tumor spread and evidence showed that autophagy and EMT are correlated. EMT is a complex process. First, a wide range of factors including cytokines and growth factors (such as growth factors with affinity for receptor tyrosine kinases and transforming growth factor (TGF)-β1) [43, 44], morphogenetic signals (namely Hedgehog, Notch, and Wnt signaling) [45], and post-transcriptional gene regulator microRNAs [46]activate EMT transcription factors such as Snail(Snail1), Twist1/2, ZEB1/2 and Slug(Snail2). Subsequently, EMT transcription factors drive EMT. EMT allows cells to show mobility and invasiveness, which is related to tumor progression, metastasis and drug resistance [47, 48]. In human hepatocellular carcinoma (HCC), HepG2 cells can induce autophagy by EMT and TGF-β1/SMad3 [49]. TGF-β1 was found to increase the invasion of bile duct cancer cells through the EMT mechanism [50]. Nitta and colleagues indicated that autophagy occurred in nutrient deficiency-induced bile duct cancer cells, and immunohistochemical staining of CCA tissues showed that the expression of autophagy-associated protein Ambra1 was positively correlated with the expression of Snail, one of the major transcription factors of EMT. They found that inhibition of autophagy by chloroquine (CQ) attenuated the invasion of CCA cells under nutrient deficiency conditions and also reduced the invasion of CCA cells induced by TGF- β1 [51]. Similarly, in bladder cancer cells exhibiting nutrient deficiency, EMT induces autophagy through the TGF-β1/Smad3 signaling pathway and promotes the invasion of bladder cancer cells [52].

The role of epigenetic modifications such as DNA hypermethylation, microRNA and histone modification in the pathophysiology of CCA has attracted more and more attention [53–55]. It has been suggested that epigenetic abnormalities play an important role in the development of CCA [53] and also can regulate autophagy [56]. DNA methylation is a reversible chemical modification of promoter CpG island cytosine catalyzed by DNA methyltransferase family, which leads to gene inactivation through transcriptional inhibition [57, 58]. DNA methylation mediated silencing of tumor suppressor genes can often be seen in CCA. Wang et al. found that IDH1/2 DNA hypermethylation (called mutant IDH1/2) was found in 10% of iCCA. This mutant IDH1/2 had higher expression level of p53 and longer recurrence time and survival time than wild-type IDH1/2, suggesting that hypermethylated CCA may represent a different molecular subclass with a better prognosis [59]. Some studies have found the relationship between autophagy inhibition and histone methylation in IDH mutant gliomas [60], which provides a new treatment approach for CCA with autophagy inhibitors. Many microRNAs, such as miR-21, miR-200b and miR-29b, are up-regulated or down-regulated in CCA. They are not only biomarkers of CCA, but also therapeutic targets [61]. MicroRNAs are also involved in autophagy regulation in tumors and have been shown to alter the levels of several key proteins in the autophagy pathway, including Beclin1, LC3, ULK2, ATG4 and ATG9 [62, 63]. Studies found that the expression of miR-124 was down-regulated in CCA. miR-124 induces autophagy-related cell death in CCA cells by down-regulating the anti-apoptotic factor Bcl-2 and activating the autophagy-promoting protein Beclin1 by inhibiting STAT3 [64]. Overexpression of histone deacetylase 1 (HDAC) in iCCA has been reported to be associated with lymph node metastasis, vascular invasion and low survival [65]. Other HDACs has also been found in CCA. HDAC6 participates in the autophagy degradation pathway. HDAC6 inhibitors can inhibit autophagy in multiple myeloma and neuroblastoma. In a mouse model of colon cancer, the HDAC6 inhibitor was used in combination with bortezomib to inhibit tumor growth in vivo. Gradilone and his colleagues found that the overexpression of HDAC6 in CCA can promote the shortening of primary cilia and the malignant transformation of normal bile duct cells. Inhibiting HDAC6 can restore cilia expression and inhibit tumor growth [66].

## The role of autophagy key proteins in CCA

Although the pathological role and regulatory mechanism of autophagy in the development of CCA are not fully understood, some recent reports have revealed important autophagy key proteins in CCA, emphasizes the significance of these autophagy-related proteins in CCA, and provided therapeutic pathway for CCA.

Beclin1 is the first identified autophagy effector of mammalian cells and a key factor in the initiation of autophagy. Studies have found that Beclin1 inactivation and autophagy defects can lead to malignant transformation of cells. The prognosis of Beclin1 is inconsistent for different tumors. When the expression of Beclin1 is different, the tumor metastasis, prognosis and survival rate are also different [12, 67, 68]. In nasopharyngeal carcinoma, highly expressed Beclin1 predicts poor prognosis [69]. In breast cancer, low expression of Beclin1 may contribute to tumor occurrence and development [68]. In CCA, studies have demonstrated the importance and prognostic value of Beclin1 [70, 71]. Dong and colleagues evaluated Beclin1 expression levels in iCCA samples. Compared with normal bile duct epithelial cells, the expression of Beclin1 was increased in most iCCA samples. In Beclin1 positive samples, low expression of Beclin1 was significantly associated with lymph node metastasis, lower overall survival and disease-free survival [70].

LC3 is another key autophagy regulator and a potential prognostic biomarker for cancer [72, 73]. It contains two isoforms: one is the soluble form LC3B(LC3B-I), the other is the lipidized form LC3B(LC3B-II). When LC3-I is converted to LC3-II, it indicates autophagy induction. During autophagy, the soluble form is transformed into the lipidized form and becomes a part of the autophagy membrane [74]. In a study, Chen et al. demonstrated for the first time that LC3B is an independent predictive biomarker for overall survival and disease-free survival of iCCA, and that high expression of LC3B indicates poor tumor differentiation, early recurrence and short long-term survival [75].

In CCA, FOXO1 is associated with autophagy flux. FOXO1 is an important transcription factor with a highly conserved DNA binding domain and is widely expressed in the spleen, liver and lung [76–78]. He et al. demonstrated for the first time that FOXO1 exists in autophagy regulation and can cause oxidative stress in human CCA cells by impairing autophagy flux, suggesting that FOXO1 may be a potential therapeutic target for CCA [79].

Death associated protein kinase 1 (DAPK1), one of Ser/Thr kinase family, is an important tumor suppressor. It mainly inhibits tumor cell growth by inducing autophagy and apoptosis [80]. We know that Beclin1 is a tumor suppressor, which affects the stability of p53 by affecting deubiquitination enzymes. DAPK1 inhibits tumor proliferation by different mechanisms, including upregulation of p53 function, etc [81]. As an autophagy inducer, however, how to inhibit tumor remains unclear.

## Autophagy modulator in CCA

### Autophagy activators

The procedure of autophagy is activated and regulated by numerous activator and mechanisim. Following are some activators for iniation of autophagy (Table 1).

**Table 1:** The effect of autophagy modulators on the development of CCA.

Autophagy regulator	Mechanism of action	Effects on CCA	Reference
Autophagy activators			
Piperlonguminea (PL) (natural product derived from piper longum plant)	Induces high levels of ROS production via multiple pathways	Induces apoptosis and autophagy through ROS-activated Erk signaling	[131]
Pterostilbene (a natural methoxylated analogue of resveratrol)	Induces cell cycle arrest and non-caspase-dependent autophagy death	Inhibits the proliferation of cells through autophagy activation (Increased the expression of ATG5, Beclin1, LC3-II and inhibited the expression of P62 in vitro)	[147]
Pristimerin (a triterpenoid and can be isolated from Celastraceae and Hippocrateaceae)	Has multiple targets	Reduces apoptosis-related proteins Bcl-2, Bcl-XL and procaspase-3 in vitro and inhibits tumor growth in vivo	[104]
Dihydroartemisinin (DHA)	Induces ROS-mediated ER stress by activating DAPK, promotes the destruction of Beclin1-Bcl-2 complex	Casparase-dependent apoptosis and autophagy-dependent death pathways	[25]
ABTL0812 (a kind of small molecule drugs)	Induces ER stress and TRIB3-mediated Akt/mTOR axis inhibition, leading to cytotoxic autophagy	Induces cytotoxic autophagy of cells by inducing endoplasmic reticulum stress	[7, 38]
Phenformin (a biguanide compound)		Upregulates autophagy-related genes ATG5, ATG7 and Beclin1 in vitro and inhibits tumor growth in vivo	[101]
ABC294640 (a novel specific inhibitor of Sphk2)	Inhibits STAT3 phosphorylation, induces caspase-dependent apoptosis, induces autophagy	Enhances cytotoxicity and apoptosis, inducing protective autophagy	[136]
Autophagy inhibitors			
Capsaicin (the main pungent component in chili pepper)	Interferes with NF-κB and AP-1 signal transduction	Reverses Beclin1 and ATG5 upregulation and activates the PI3K/AKT/mTOR pathway to inhibit 5-Fu-induced autophagy	[30, 92]
Chloroquine (CQ) (an antimalarial drug)	Accumulates a large number of degraded proteins and induces ER stress	Reduces cell invasion and TGF-β 1-induced cell invasion under starvation	[34, 52, 98]
Hydroxychloroquine (HCQ)	Inhibits the membrane fusion between autophagosome and lysosome	Increases the number of apoptotic cells and the expression of apoptosis-related proteins and blockes the G1 phase	[69, 86, 106]
Resveratrol (a natural polyphenolic antitoxin)	Inhibits FOXO1 acetylation and impairs the binding of FOXO1 and ATG7	Induces apoptosis by oxidative stress and mitochondrial dysfunction	[118]
Oblongifolin C (OC) (a natural small molecule compound)	Inhibits autophagosome-lysosomal fusion and autophagic degradation	Induces apoptosis and mitochondrial dysfunction	[90, 149]
Salinomycin (Sal) (a polyether antibiotic)	Interferes with WNT signaling	Leads to the accumulation of dysfunctional mitochondria and the increase of ROS	[78]

#### Piperlongumine (PL)

PL, a natural product derived from piper longum plant, induces high levels of ROS production [82]. It passes through NF-κB, P38/JNK and other signaling pathways lead to apoptosis or autophagy in colon cancer, breast cancer, pancreatic cancer, kidney cancer, ovarian cancer, head and neck cancer and prostate cancer [83, 84]. Chen et al. found that PL also induced autophagy in CCA cells. In an in vitro experiment, they found that PL induced autophagy and apoptosis of HuCCT-1 cells via ROS-activated Erk signaling, and autophagy was inhibited when the Erk signaling pathway was inhibited [85]. This is the first report to demonstrate that PL can treat CCA by inducing cell autophagy. However, the specific efficacy has not been thoroughly studied, and a large number of studies are still needed if PL is applied to treat CCA in the future.

#### Pterostilbene

Pterostilbene is a natural methoxylated analogue of resveratrol, which has higher natural utilization, cell absorption, better lipophilicity, and a longer half-life than resveratrol [86]. Rimando et al. demonstrated that it has anti-inflammatory, anticancer and antioxidant biological effects [87]. Many studies have reported that pterostilbene has autophagy induction properties for various tumors [88–91]. In one study, after treating CCA cells with pterostilbene, it was found that pterostilbene did not induce cell apoptosis, but inhibited the cell proliferation by activating autophagy. It increased the expression of ATG5, Beclin1, LC3-II and inhibited the expression of P62 in vitro, which suggested the enhancement of autophagy activity. When CCA cells were treated with autophagy inhibitor 3-MA, autophagy induction and anti-tumor activity were inhibited [86].

#### Pristimerin

Pristimerin is a triterpenoid and can be isolated from Celastraceae and Hippocrateaceae. Sun et al. found that pristimerin significantly reduced the expression of apoptoses-related proteins procaspase-3, Bcl-2 and Bcl-XL, increased the expression of Bax, and promoted Beclin1 activation and autophagy initiation in vivo and in vitro [92]. In addition, they found that when the eCCA cell line QBC939 cells and the iCCA cell line RBE cells were treated with different concentrations of pristimerin, the inhibitory effect of this compound on QBC939 cells was stronger than that on RBE cells. The reason for the selectivity needed to be further studied [92].

#### Dihydroartemisinin (DHA)

DHA is an anti-malaria drug. Consistent with previous reports, DHA can increase the expression of autophagy-related genes such as ATG12 and BNIP3 while decreasing mTOR expression [93, 94]. Previous studies found that DHA killed CCA cells mainly through two pathways: caspase-dependent apoptosis and autophagy-dependent death pathways. Ser/Thr kinase DAPK is the main common mediator of these two pathways, which can regulate both apoptosis and autophagy [95–97]. Thongchot and colleagues reported that DHA induced ROS-mediated ER stress by activating DAPK, promoted the destruction of Beclin1-Bcl-2 complex and induced the death of CCA cells, thereby activating autophagy. Interestingly, DHA is only mildly toxic to normal bile duct cells but cytotoxic to cancer cells. In CCA cells lacking Beclin1, autophagy activation is blocked, which shows that DHA also needs Beclin1 activation [98].

#### Rapamycin and its analogues

Rapamycin and its analogues have been used as cancer therapeutic agents. One of their potential therapeutic mechanisms is the induction of autophagy, which may become a new molecular target for CCA therapy [99, 100]. The PI3K/Akt/mTOR pathway is a crucial signaling cascade regulating cell growth, proliferation and apoptosis [101]. Mammalian rapamycin target protein (mTOR) is a downstream effector of PI3K/Akt signaling pathway and is considered to be a therapeutic target for many diseases. The first definite mTOR inhibitor is rapamycin (sirolimus), also widely known as an autophagy inducer. Rapamycin and its analogue everolimus (RAD001) showed anti-tumor activity in previous studies [102–104]. In a study, rapamycin increased the sensitivity of PTEN mutant testicular cancer cells to radiotherapy by inducing autophagy [102, 105]. In a phase IB clinical study, RAD001 inhibited the growth of endometrial cancer cells and had synergistic effects with other anticancer drugs [106].

#### ABTL0812

ABTL0812 is a kind of small molecule drugs with anti-tumor activity. Currently, some phase 2 clinical trials are evaluating the efficacy of this drug in endometrial cancer and lung cancer [107, 108]. Previous studies have shown that ABTL0812 induces AKT-MRORC1 inhibition and autophagy-mediated tumor cell death by promoting the expression of TRIB3, a pseudokinase that binds and prevents AKT activation by upstream kinases PDPK1 and MTORC2 [107, 109]. In CCA, ABTL0812 has inhibitory effect on CCA cells. It can induce cytotoxic autophagy of CCA cells by inducing ER stress and TRIB3 mediated inhibition of Akt/mTOR axis [109, 110]. Similar to DHA, normal bile duct cells can survive at the concentration of ABTL0812 and are fatal to CCA cells, suggesting that ABTL0812 may provide a new and safe method for CCA treatment.

#### Phenformin

Phenformin is a biguanide compound, similar to metformin, used to treat type 2 diabetes [111]. In a study, Hu and his colleagues found that when CCA cells were treated with phenformin, autophagy-related genes ATG5, ATG7 and Beclin1 were up-regulated and cell death was increased, suggesting that phenformin can inhibit the growth of bile duct cancer cells by inducing autophagy in CCA cells [112]. Therefore, phenformin is expected to be a treatment for CCA.

#### ABC294640

Sphingosine kinase (Sphk) is a lipid kinase with a carcinogenic effect [113–117]. Studies have shown that Sphk subtype Sphk2 can produce mitogenic lipid sphingosine-1-phosphoric acid (S1P) to promote the growth of CCA [118]. ABC294640 is a novel specific inhibitor of Sphk2. Ding and colleagues found that ABC294640 can induce autophagy in CCA cells and can enhance the cytotoxicity and apoptosis induced by ABC294640 through the use of autophagy inhibitors bafilomycin A1 and chloroquine, thus inducing protective autophagy [119].

#### Urolithin A

The polyphenols ellagitannins and ellagic acid, which are found in nature, produce urolithin A (UA), a substance with anticancer action against a variety of cancers [120].

Studies have shown that UA can have several effects on gastric cancer (GC) cells in vitro. It can restrict the movement and invasion of these cancer cells, promote cell death (apoptosis), and induce autophagy by influencing the phosphatidylinositol-3-kinase/protein kinase B/mammalian target of rapamycin (PI3K/AKT/mTOR) pathway [120–122]. UA is expected to be used for GC treatment.

### Autophagy inhibitors

There are also numerous inhibitors of autophagy procedure (Table 1).

#### Capsaicin

Capsaicin, the main pungent component in chili pepper, can inhibit human liver cancer, breast cancer and other malignant tumors [123, 124]. Among the natural compounds that can inhibit CCA autophagy, capsaicin is the only one that inhibits autophagy by activating mTOR. Existing studies have shown that capsaicin negatively regulates cell survival, adhesion, inflammation, differentiation and growth by interfering with nuclear factor-κB (NF-κB) and activator protein-1 (AP-1) signal transduction [125]. In CCA, capsaicin can reverse Beclin-1 and ATG5 upregulation and activate the PI3K/AKT/mTOR pathway to inhibit 5-Fu-induced autophagy [126]. Capsaicin in combination with 5-Fu also enhanced the sensitivity of QBC939 cells in vitro and in vivo [126], suggesting that capsaicin is promising as a new treatment option for CCA patients in combination with chemotherapy agents. However, the mechanism of the combination of capsaicin and chemotherapy drugs still needs more in-depth research.

#### Chloroquine (CQ)

CQ is an antimalarial drug that blocks the combination of lysosomes and autophagosomes by changing the acidic environment of lysosome, so as to accumulate a large number of degraded proteins and induce ER stress [127–129]. In CCA model, CQ could reduce CCA cell invasion and TGF-β 1-induced CCA cell invasion under starvation [129]. In addition, CQ can also improve the sensitivity of drug-resistant bile duct cancer cells to chemotherapy drugs [127]. Qu et al. compared the activity of QBC939 cells and HepG2 cells treated with CQ and found that the activity of QBC939 cells was more significantly inhibited[127]. The cell activity of CQ in combination with cisplatin was much lower than that of CQ alone. They further confirmed that the levels of autophagic proteins P62 and LC3-II/I in QBC939 cells were significantly increased after treatment with CQ, suggesting that QBC939 cells had high autophagy ability. This provides a new therapeutic idea for CQ as an autophagy inhibitor to inhibit autophagy and thus inhibit CCA cell growth in the future.

#### Hydroxychloroquine (HCQ)

HCQ acts similarly to chloroquine, both of which were initially used only as an adjuvant in combination with other antineoplastic agents [130, 131]. It was later found that HCQ or CQ alone can also produce good anti-tumor effects. HCQ induced apoptosis of CCA cells by upregulating ROS. With the addition of ROS scavger GSH, HCQ mainly inhibits the final stage of autophagy, namely the membrane fusion between autophagosome and lysosome [132, 133]. According to the results of Chen et al., when CCA was treated with IC50 concentration of HCQ, the number of apoptotic cells and the expression of apoptotic-related proteins in CCA cells increased, and G1 phase was blocked. At the same time, autophagy continued smoothly upstream, and the expression of autophagy-related proteins increased [134]. These results suggest that HCQ can inhibit the proliferation of bile duct cancer cells and induce apoptosis by inhibiting the accumulation of ROS induced by autophagy. However, this is only shown to be effective in animals, and the detailed mechanism in vivo needs to be further studied.

#### Resveratrol

Resveratrol is a natural polyphenolic antitoxin found in grapes. It has a variety of benefits for humans, especially in cardiovascular aspects. Previous studies have shown that resveratrol can induce autophagy-mediated cell death in gastric cancer and leukemia cells [135, 136]. In CCA, resveratrol inhibits autophagy by inhibiting FOXO1 acetylation, impairing the binding of FOXO1 and ATG7, leading to apoptosis [79]. He et al. found in vitro that one of the mechanisms of resveratrol induced apoptosis of QBC939 cells is that the inhibition of autophagy leads to oxidative stress and mitochondrial dysfunction (MTD) [79].

#### Oblongifolin C (OC)

OC is a natural small molecule compound extracted from *Garcinia yunnanensis*. Previous studies have demonstrated that OC is a novel inhibitor of autophagy flux, which can inhibit autophagosome-lysosomal fusion and autophagic degradation [137]. However, the effect of OC on CCA remains to be clarified. Zhang and colleagues treated CCA QBC939 cells with OC, and found that OC could inhibit autophagy and promote mitochondrial dysfunction (MtD) by blocking the fusion of autophagosome and lysosome, thus inducing apoptosis of CCA cells [138].

#### Salinomycin (Sal)

Sal is a polyether antibiotic, which has anti-tumor effects in liver cancer, leukemia, colon cancer and other tumors [139–141]. It has been proved that Sal can inhibit autophagy and play an anticancer role in CCA. Klose et al. treated CCA cells with different concentrations of Sal and found that the autophagy flux of CCA cells was inhibited in a dose-dependent manner. They also found that Sal also led to the accumulation of dysfunctional mitochondria and the increase of ROS after inhibiting autophagy in CCA cells, suggesting that it can influence autophagy and lysosomal fusion just as CQ does [142].

## Discussion

Autophagy is a closely coordinative process that can maintain average cell growth and metabolism, prevent energy depletion after stress, and sustain protein and nutrient balance [143, 144]. Due to the multiple regulatory points in autophagy, studying the precise molecular mechanisms between autophagy and tumor is challenging. Due to the multiple regulatory sites of autophagy, it is challenging to study the precise molecular mechanisms between autophagy and tumors. In CCA, the current study seems to have little understanding of the relationship between autophagy and CCA. Although preclinical studies on targeted autophagy in the treatment of CCA have some results, its clinical studies are still uncertain. We can now take a little inspiration from studies of lung cancer, breast cancer and other cancers. The combination of autophagy and immunotherapy has been found in non-small cell lung cancer [145]. Autophagy plays an important role in adaptive immune responses. When ATG5 is lost, it not only damages autophagy, but also disrupts B cell development and T cell homeostasis [146, 147]. Combining autophagy regulation with immunotherapy will be a key area of future research. In clinical studies, targeted therapy with Traditional Chinese medicine can also regulate autophagy. Furanodiene is a natural compound isolated from the Rhizoma Curcumae. Xu et al. pointed out that it can stimulate ER stress and induce autophagy and apoptosis of lung cancer cells, indicating that autophagy can regulate the treatment of Traditional Chinese medicine [148]. In clinical treatment, Chinese medicine treatment can also regulate autophagy. In lung cancer, Chinese medicine affects the pro-death activity and protection of autophagy of lung cancer cells. Isodeoxycholin (ESI) shows potential anticancer effect [149]. After ESI treatment, autophagy markers such as ATG3, LC3-II and Beclin1 in lung cancer cells increased significantly, and therapeutic effects were produced through various channels. *Marsdenia tenacissima* (MTE) is a TCM that has been used to treat asthma, rheumatism, and tracheitis for thousands of years. MTE may induce apoptosis and autophagy inhibition coinstantaneously in lung cancer cells. Extracellular signal-regulated kinases (ERK) activation is partially linked with apoptotic and autophagic cell death after MTE treatment. Therefore, the mechanism of MTE induces apoptosis and suppresses autophagy via ERK activation [150]. Bu-Zhong-Yi-Qi Decoction (BZYQD) as a potential anti-tumor TCM. ROS accumulation may activate apoptosis and autophagy via oxidative stress [151].

The combination of autophagy and immunotherapy has been found in non-small cell lung cancer [145]. In contrast, autophagy has been well studied in the treatment of breast cancer. The effect of autophagy on breast tumor cells mainly depends on the stage of breast cancer development and previous treatment in the treatment process. Commonly used chemotherapeutic agents induce sustained autophagy and mediate autophagic cell death pathways. At this point, the use of the novel mTORC1/2 kinase increases autophagic flux and thus increase cytotoxic effects [152]. On the contrary, forced autophagy after chemotherapy may induce drug resistance, and tumors are prone to relapse. Therefore, drugs that disrupt autophagy mechanisms can be selected. In conclusion, the treatment strategies and directions for breast cancer at different stages are quite different.

Combined with the study of CCA, autophagy plays a multifaceted role in it. Even in different subtypes of CCA, autophagy has an environmentally dependent or conflicting role, suggesting a need to integrate the stages of disease development with autophagy characteristics. Given the heterogeneity of CCA and the potential need for long-term use of autophagy modulators during treatment, it is necessary to develop new biomarkers to further monitor autophagy status in patients with CCA and adjust medication according to this autophagy status. At present, various autophagy modulators for the treatment of CCA are still in the stage of preclinical research. Using preclinical models can further reveal the specific mechanism of autophagy in CCA. Comparing the autophagy levels of different mutated CCA cells can help us study how the mutation further regulates autophagy, and which subtype of patients with CCA will have a higher therapeutic effect if different autophagy modulators are applied. Therapies that combine autophagy regulation with immunotherapy, chemotherapy, and targeted therapies appear to be promising strategies for the treatment of CCA in the future.
